# Study on Phase Change Materials’ Heat Transfer Characteristics of Medium Temperature Solar Energy Collection System

**DOI:** 10.3390/ma17215159

**Published:** 2024-10-23

**Authors:** Tianqi Wang, Yingai Jin, Firoz Alam

**Affiliations:** 1 National Key Laboratory of Automotive Chassis Integration and Bionics, Changchun 130022, China; wangtq23@mails.jlu.edu.cn (T.W.); jinya@jlu.edu.cn (Y.J.); 2 College of Automotive Engineering, Jilin University, Changchun 130022, China; 3 School of Engineering (Aerospace, Mechanical and Manufacturing), RMIT University, Melbourne, VIC 3000, Australia

**Keywords:** phase change material, energy storage, solar energy utilisation, energy saving

## Abstract

Within the next five years, renewable energy is expected to account for approximately 80% of the new global power generation capacity, with solar power contributing to more than half of this growth. However, the intermittent nature of solar energy remains a significant challenge to fully realizing its potential. Thus, efficient energy storage is crucial for optimizing the effectiveness and dependability of renewable energy. Phase-change materials (PCMs) can play an important role in solar energy storage due to their low cost and high volumetric energy storage density. The low thermal conductivity of PCMs restricts their use for energy storage, despite their immense potential. Hence, the primary goal of this study is to experimentally investigate the energy storage capacity of two blended phase-change materials (paraffin and barium hydroxide octahydrate) through integration with a medium-temperature solar heat collection system. The experimental findings reveal that the blended PCMs possess the highest cumulative charge fraction (0.59), energy capacity, and low energy loss compared to each PCM alone. Furthermore, the phase change storage tank achieves higher heat storage (27%) and exergy storage efficiency (18%) compared to the stored tank water without any PCMs. The blended PCMs enhanced their performance, exhibiting improved interaction and excellent thermal storage properties across a range of temperatures, offering an opportunity for the design of an energy-efficient, low-cost storage system.

## 1. Introduction

Experts predict a steady rise in the demand for thermal energy, which currently accounts for 50% of global energy consumption [1]. In light of this growing demand, the development and implementation of hybrid renewable energy technologies such as solar and wind have become critical [2,3]. These technologies are vital in reducing carbon footprints and fostering sustainable development, driven by economic growth, the necessity for sustainable infrastructure, and a deepening comprehension of technology’s impact on climate change [4]. However, the intermittent nature of renewable energy poses a significant challenge to their widespread application. To mitigate this, efficient and low-cost thermal energy storage development is crucial for wider acceptability and application [5].

Stakeholders, including government policymakers and researchers, are actively working to reduce carbon emissions as carbon budgets are rapidly depleting. This effort has led to a significant shift towards renewable energy and electrification, with a particular focus on wind and solar energy development. In line with this transition, there is a noticeable change in the structure of energy demand. Additionally, the use of new materials for energy storage is gaining prominence as a strategy to further decrease carbon emissions. This study specifically addresses the role of solar collector systems and PCMs in the efficient storage and utilization of solar energy resources, highlighting their potential to contribute to carbon emission reduction.

**Figure 1:**
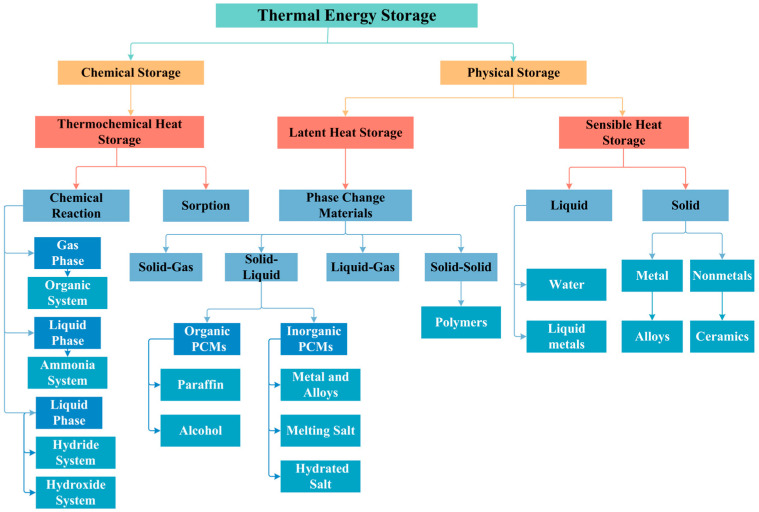
Classification of thermal energy storage materials.

The current state of the art in thermal storage technology is highlighted in Figure 1. Among various thermal energy storages (TESs), latent heat storage (LHS) technology based on phase change materials (PCMs) has gained widespread attention from researchers in recent years due to its high energy storage capacity, simplicity of operation, and enormous potential, playing a key role in the development of sustainable energy [6]. PCM is a material used to store or release thermal energy during phase transitions, and even after hundreds of thousands of phase transition cycles, PCMs maintain their latent heat of phase transition unchanged [7]. PCMs are mainly classified into three types according to their chemical structures: (a) inorganic PCMs, (b) organic PCMs, and are divided into four categories according to the different phase change processes, namely (i) solid–liquid, (ii) solid–solid, (iii) solid–gas, and (iv) liquid–gas [8]. At present, PCMs continue to face challenges such as leakage and poor thermal conductivity. Consequently, shape-stabilized phase change materials, which are based on porous carriers, have emerged as innovative solutions to address these issues [9,10]. For the selection of PCM, it is crucial to opt for materials that align with the overall system requirements. While there is a diverse range of PCMs available, not all are suitable for each and every application. When making choices, several factors need to be considered, such as (1) ensuring the phase change temperature falls within the practical working range, (2) possessing a high latent heat storage capacity, (3) exhibiting high thermal conductivity, (4) maintaining stable chemical and thermal properties, (5) being non-toxic, non-corrosive, and environmentally friendly, (6) being cost-effective and readily available, (7) undergoing a relatively small volume change during the phase transition process, and (8) experiencing no or low undercooling [11]. An ideal PCM should meet a comprehensive set of requirements, encompassing thermal storage performance and the specific physical and chemical properties demanded by particular application systems. These requirements include a high enthalpy of phase change per unit volume, exceptional thermal conductivity, substantial specific heat capacity, good crystallinity, a rapid crystallization rate, and stable chemical properties [12,13].

**Figure 2:**
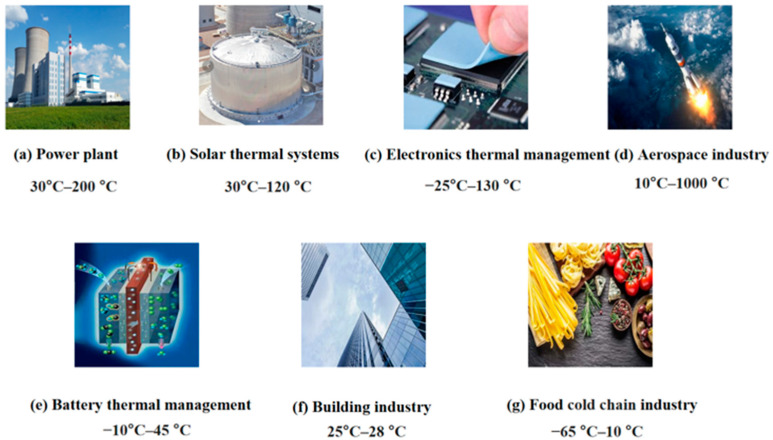
Industry application areas of PCM and their temperature range.

The industry sector accounts for approximately 35% of the world’s total energy demand [14]. The industrial sector releases a significant amount of waste heat directly into the atmosphere that can otherwise be repurposed. As shown in Figure 2, PCMs have great potential in different temperature ranges and in different industrial applications, including but not limited to power plants [15], solar thermal systems [16], electronics thermal management [17], aerospace industry [18], battery thermal management [19], thermal management of buildings [20], and the food industry [21]. The application of PCMs across these diverse fields underscores their effectiveness in optimizing thermal management and energy efficiency. The temperature range for these applications is diverse, with most being utilized, at least partially, in the medium-temperature range. Medium- and high-temperature PCMs have diverse applications, but medium-temperature PCMs offer enhanced safety and greater flexibility in equipment selection compared to high-temperature options. This paper focuses on the study of medium-temperature solar systems.

Generally, PCMs are the common energy storage medium for solar systems due to their high thermal storage density, isothermal nature of the storage process within a certain temperature range, and easy control [22]. The thermal properties of PCMs have garnered significant research interest, as they are critical considerations for PCM selection. Hydrated salt and paraffin wax are the most common phase change materials [23,24].

Table 1 shows some basic properties of selected salt-hydrate materials. The selection covers PCMs over a wide range of phase change temperatures, which exhibit consistently high latent heat capacities (in the range of 110 kJ/kg to 300 kJ/kg). Additionally, other important physical properties, e.g., density, specific heat, thermal conductivity, etc., which are essential for TES applications, are also presented in the table.

Paraffin, a unique material, is mainly composed of n-alkanes, while the rest are isoalkanes and cycloalkanes. It has a relatively high latent heat, ranging from 200 to 220 kJ/kg, and can melt at 47 °C to 64 °C, making it useful as PCM over a wide temperature and phase change range. Paraffin can also undergo phase transition without supercooling, with good chemical stability and no phase separation. Industrial grade paraffin, such as PCM, is the most cost-effective, feasible, and can be used widely. Barium hydroxide octahydrate (BHOH), chemical formula Ba(OH)_2_·8H_2_O, is a common and frequently used inorganic hydrated salt phase change energy storage material, with a relative molecular mass of 315, a melting point of 78 °C, a latent heat of phase change of 267 kJ/kg, and a specific heat capacity of 1.17 kJ/(kg·K). BHOH is the inorganic crystalline hydrate with the high heat storage density in the phase change temperature range of 0–100 °C. Paraffin and BHOH offer significant application advantages in energy storage systems. Zhang et al. [29] explored the performance of paraffin mixtures over 10,000 thermal cycles, finding that the decay in the heat of fusion is only 9.1%, with the melting point decay being negligible. These results suggest that paraffin is a highly promising PCM for solar systems. Rahim et al. [30] conducted experiments using a specialized collector system to heat paraffin, demonstrating that 2 kg of paraffin could absorb 400 min of solar energy, achieving a maximum heat storage of 730 kJ. The paraffin impressively retained 621 kJ of heat even after cooling for 6 h, demonstrating excellent heat retention properties. Xiao et al. [31] developed a shape-stable composite PCM combining BHOH with modified expanded graphite (MEG). This composite demonstrated good thermal reliability. Additionally, the incorporation of MEG notably reduced the supercooling temperature of BHOH from 13 °C to 2.4 °C. Zhang et al. [32] utilized eutectic salts with lower melting points than conventional types as both a thermal conductive fluid and an energy storage medium at solar tower power plants in Nottingham, UK, and Dezhou, China. Their study revealed that employing these point eutectic salts extended the annual operating time of the Dezhou power station by 75 days and that of the Nottingham power station by 33 days. Additionally, the mixing of pure salts was identified as a simple method to enhance the efficiency of solar power plants.

Table 2 presents the most important findings from past research on integrating PCM in solar collector systems [33,34,35,36,37,38,39,40,41]. As described in Table 2, the integration of PCM into solar energy systems encounters numerous challenges. Research on PCMs remains confined to specific material types, with limited exploration into PCM shapes and sizes. Furthermore, the potential issues associated with PCM integration into solar energy systems are frequently overlooked, and there is a deficiency in comparisons with other solar energy system types. Addressing these issues is essential for optimizing the performance and viability of PCM-enhanced solar energy storage systems. So, in this paper, it was decided to jointly experiment with phase change energy storage tanks using different types of phase change materials for thermal storage to provide a solution for sustainable energy.

An effective thermal design for solar water heating systems enhances the use of solar thermal energy [42]. However, challenges such as fluctuating solar energy and the insufficient heat storage density of fluid water limit the optimization [43]. Latent heat storage tanks offer the benefits of traditional thermal storage systems while increasing the volume of hot water available and reducing the overall system size. The optimization and application of thermal storage systems for solar hot water, as well as their efficient uses, are currently a prime research focus worldwide [44,45].

Al-Hinti et al. [46] performed experiments with a phase change energy storage tank using paraffin as the phase change material and observed the system after stopping heating for 14 h. They found that the water temperature was 14 °C higher compared to a system without PCM and that the temperature could be maintained above 45 °C for an extended duration. De Gracia et al. [47] discovered that the experimental tank utilizing TH58 as the phase change material had an exothermic capacity 3.5 kJ greater than that of a tank without PCM. Mazman et al. [48] conducted a heat storage study using three types of binary organic composite phase change materials (paraffin-stearic acid, paraffin-palmitic acid, and stearic acid-myristic acid) in a 150 L hot water tank. They added these materials to the upper part of the tank to assess improvements in heat storage. The study found that all three composite phase change materials enhanced the tank’s heat storage capacity. Among them, the paraffin-stearic acid composite PCM showed the most significant improvement in both storage and heat release performance. It can provide 14–16 L of hot water at 3–4 °C higher than a standard tank within 10–15 min, improving the overall collector efficiency of the solar domestic hot water system by 74%.

The studies reviewed above indicate that the phase change energy storage tank possesses effective heat storage capacity. However, the combined effect of two phase materials on the transition temperatures for medium-temperature solar thermal storage has not been well studied and reported in the public domain. Therefore, the primary goal of this study is to experimentally investigate a medium-temperature solar thermal storage system utilizing two PCMs with significantly different phase transition temperatures. Furthermore, the study aims to analyze and comprehend the distinct phase transition characteristics of each PCM and evaluate their respective and combined contributions to the energy storage efficiency.

## 2. Material and Experimental Setup

### 2.1. Selection and Encapsulation of PCM

**Figure 3:**
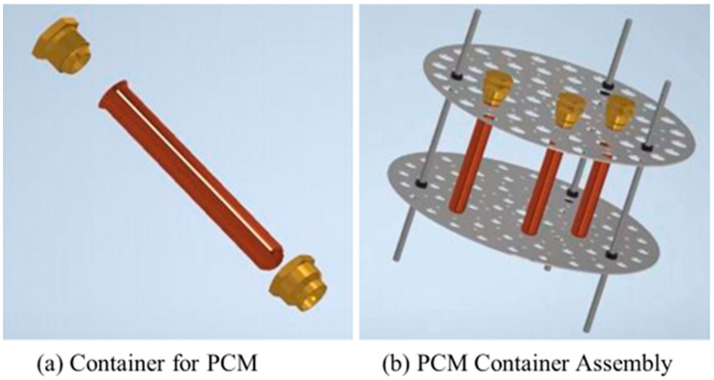
Schematic of phase change material encapsulation.

To fully harness solar energy, it is essential to utilize PCMs with distinct temperature ranges. Paraffin stands out as a widely used PCM due to its cost-effectiveness, thermal stability, availability, and eco-friendliness. On the other hand, BHOH offers high thermal storage density, low volume change during phase transitions, and significant thermal conductivity at a low cost. So, paraffin and BHOH are chosen as the PCM. Table 3 lists the characteristics of paraffin and BHOH. During the operation, PCM absorbs heat, its temperature rises to the melting point, and it transforms from solid to liquid. Paraffin and BHOH were procured from Sinopharm Chemical Reagent Co., Ltd. in Shanghai, China. The paraffin has a melting point range of 50–52 °C with a purity of 100%, while the BHOH boasts a purity of 97%. In this experiment, both mass fraction and volume fraction are based on the total mass and volume of water and PCM in the tank. When only paraffin was used as the PCM for the experiment, the volume fraction of PCM was 1.99% and the mass fraction was 1.80%; when only BHOH was used as the PCM for the experiment, the volume fraction of PCM was 2.41% and the mass fraction was 5.11%; and when both PCMs were used, the total mass fraction of PCM was 6.87%. Paraffin and BHOH are selected as the main part of the PCM for the phase change energy storage tank, in which the phase change temperature fits the system temperature requirements. The PCM is encapsulated in the cylindrical copper tube 18 mm in diameter, 1 mm thick, and 160 mm high, as shown in Figure 3.

### 2.2. Experimental Setup

**Figure 4:**
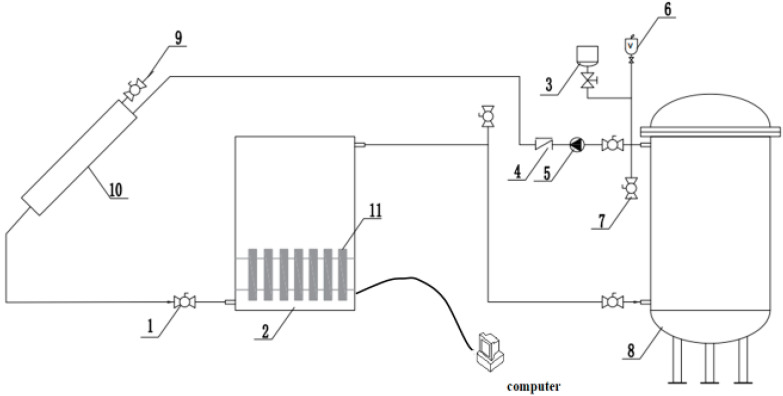
Schematic of experimental setup (inside the enclosure). 1—Hot water inlet valve; 2—Buffer tanks; 3—Pressure expansion tanks; 4—Check valve; 5—Pump; 6—Automatic exhaust valve; 7—Manual exhaust valve; 8—Phase change energy storage tank; 9—Water supply valve; 10—Solar collector; 11—Sealed copper container with PCMs, thermocouples attached to copper container. The valve next to water pump (5) is water return valve.

**Figure 5:**
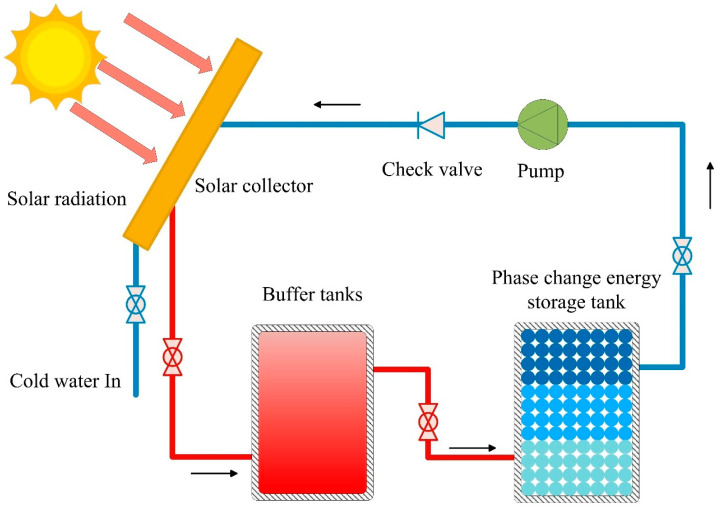
Schematic of experimental setup with solar collector. (The arrow indicates the direction of system circulation).

A schematic diagram of the experimental system, which consists of a solar collector, a PCM tank, a buffer tank, a centrifugal pump, a pressure expansion tank, and some valves, is shown in Figure 4 and Figure 5. A vacuum tube solar collector with a Fresnel lens was employed in a medium-temperature solar collector system. Dimension of the collector (L × W × H) (mm): 2025 × 1495 × 160, collector efficiency is 0.47.

**Figure 6:**
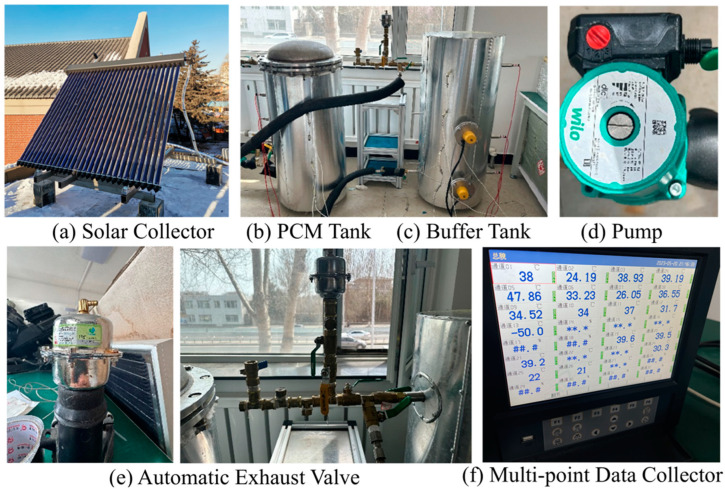
Equipment used for experimental setup and data collection.

A stainless-steel cylinder with an internal diameter of 350 mm and a height of 860 mm wrapped in insulation cotton with a thermal conductivity of 0.031 W/(m·°C) is used as a PCM tank. The thickness of insulation cotton is 80 mm. As shown in Figure 5, high-temperature water enters the tank through the inlet and exits from the bottom outlet. The temperature distribution of the PCM tank is measured using platinum resistors and thermocouples. Two of the platinum resistors are fixed at the inlet and outlet of the PCM tank. The buffer tank has an inner diameter of 350 mm and a height of 880 mm. It is constructed from stainless steel and wrapped in insulation cotton with a thermal conductivity of 0.031 W/(m·°C). The thickness of insulation cotton is 80 mm. The PCM tank and the buffer tank are equipped with temperature measurement points to measure the temperature of the tank walls and the water inside. And Figure 6 shows the actual test facility.

A circulating pump (Wilo RS15, 0–25 L/min) is used to control the water flow, as shown in Figure 4 and Figure 5. The system uses a multi-point data collector (RX8000D) from Hangzhou Meikong Automation Technology Co, Hangzhou, in China to record temperature data, and the measurement interval is set to 5 s. Three sets of welding spots are used to place the encapsulated units (copper container with PCM sealed) and shelf mesh 250 mm, 350 mm, and 450 mm vertically from the bottom of the tank, 18 encapsulated units, as shown in Figure 3, arranged evenly in each layer. Each encapsulated unit is fitted with a thermocouple to measure temperature, as shown in Figure 4(11). All temperatures have been measured in the laboratory.

**Figure 7:**
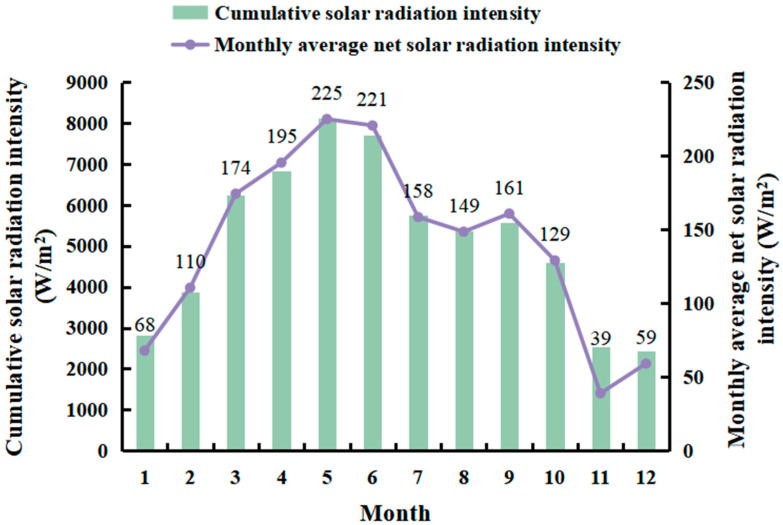
Solar radiation intensity at the experimental site in 2023 in the city of Changchun, China.

The study was undertaken in Changchun, a city in northeastern China, during sunny and warm weather in 2023, primarily from April to October. The data were taken from China’s meteorological website. To gain insight into the solar radiation at the experimental site, local meteorological data are used. The solar radiation intensity at the experimental site is shown in Figure 7.

The tank’s hollow interior is designated for housing the PCMs. In addition, four iron frames are used in the design, two of which are set up specifically to support the copper pipes. These pipes, capable of containing PCMs, are integral to the system; when filled with PCMs, they effectively transform the water tank into a phase change latent heat storage unit.

In the operational procedure, hot water is circulated through the exterior of the copper tube, across the PCM tubes. Leveraging the excellent thermal conductivity of copper, this process facilitates the rapid transfer of heat between the hot water and the PCM contained within the tube. As the PCM undergoes its transition, it effectively stores energy from the hot water. The heat exchange between the hot water and the PCM is highly efficient, ensuring optimal energy storage during the phase change. Even in the absence of sunlight, the PCM continues to undergo phase transitions. This allows the stored solar energy to be released into the water.

At the outset of the experiment, the phase change energy storage material starts at a low temperature. By absorbing solar energy, the water in the solar collector is heated and circulated using a pump, and the temperature of the PCM sample can be raised to its phase change temperature. Throughout the heating process, the material undergoes a gradual transition from a solid to a liquid, absorbing a substantial amount of thermal energy while maintaining a stable temperature. Once the phase change energy storage material reaches its phase change temperature, it initiates the absorption and storage of thermal energy. In this energy storage process, the PCM draws in thermal energy from the surrounding environment, simultaneously stabilizing the temperature and accumulating a significant amount of latent heat energy. This storage process can persist for a specific duration until the PCM either reaches saturation or the external heat source discontinues the supply of thermal energy. When it becomes necessary to release the stored thermal energy, it can be achieved by lowering the temperature of the PCM. This reduction in temperature prompts the PCM to gradually transition from a liquid to a solid, thereby releasing the previously absorbed thermal energy.

By monitoring and analyzing the energy storage system equipped with phase-change energy storage materials and comparing it with a conventional hot water storage tank, the increase in energy storage capacity and overall system efficiency can be determined. This enables the determination of the advantages/benefits associated with integrating phase change energy storage materials into solar thermal collector systems.

Before commencing the experiment, a series of some preparatory measures has been undertaken. The encapsulation of phase change materials is essential for their effective use. To encapsulate paraffin wax, it is first heated until it becomes liquid and then poured into an encapsulation container. In contrast, Ba(OH)_2_·8H_2_O, which is a solid particle, can be directly encapsulated without any preliminary processing. Concurrently, the working conditions of both the thermocouple and the multipoint recorder have been checked. To guarantee precision and reliability, the calibration on the thermocouples has been performed. These calibrated thermocouples are then strategically placed within the system and the multi-point data collector to monitor and record temperatures accurately. Upon confirming the system’s readiness, the experiment has been initiated by powering up the equipment and opening the solar inlet valve. This allowed water under pressure to flow into the solar collector. During insulation, a waiting period has been observed until the water reaches the required temperature, ensuring optimal efficiency. The next step involved transferring the heated water into the buffer tank. An excellent buffer tank should have efficient insulation, a suitable thickness of insulation, and a low heat loss coefficient. This tank has insulation and is at a stable temperature for the duration of the experiment. The solar collector’s hot water flows into the buffer tank, which is a continuous process. Furthermore, the solar collector continuously heats up the water in the storage tank so that the temperature of the water reaches the target temperature. Under good weather conditions, it can be heated quickly to the desired temperature, and the system automatically replenishes the water to the collector. Once sufficient water has been heated, hot water can flow into the PCM tank by opening the outlet valve of the buffer tank. During this process, the temperature of the water is recorded by thermocouples into a multipoint recorder, after which the data are recorded and exported for subsequent analysis.

The measuring device employed in this study is the PT1000 sensor, which enables a wide temperature measuring range spanning from −200 °C to 420 °C. With a measuring accuracy of ±0.5 °C and an uncertainty of 1 K, this sensor ensures precise and reliable temperature measurements across the spectrum of temperatures.

## 3. Methods of Analysis

Accurate evaluation and analysis of the thermal properties of materials are essential in the study of phase change energy storage systems. The study here employs a combined approach integrating energy and exergy analyses. This integrated method not only offers a scientific foundation for material selection but also establishes a basis for optimizing the design of the energy storage system. Ultimately, the goal is to improve the practical advantages of phase change energy storage technology. The ultimate objective is to find a heat storage system that can store higher-grade thermal energy and has fewer heat losses. In this section, the whole energy storage tank is further analyzed using energy and exergy.

### 3.1. Energy Analysis

Energy analysis allows us to quantify changes in a system’s energy, providing insights into efficiency and heat management during the transfer process. This understanding can lead to improved design and operational strategies.

For the energy loss of the tank $Q_{\mathrm{loss}}$, it can be expressed as Equation (1), which is equal to the energy released by the exothermic process of the whole system. Energy loss typically refers to the heat that escapes during the charging (melting) and discharging (freezing) processes. Equation (2) presents the useful energy stored in the system. In reference [50], a new parameter, Cumulative Charge Fraction (CCF), defined as the ratio of cumulative heat transferred to the storage tank up to time (*t*) to the maximum storage capacity of the PCM tank, is introduced and is given by Equation (3). With this coefficient, it is possible to compare to some extent the energy stored in different PCM systems. The higher the value, the more useful energy is stored in the system.

(1) $$Q_{\mathrm{loss}}=m_{w}C_{p,w}(T_{\max,w}-T_{t,w})$$

(2) $$Q_{\mathrm{use}}=m_{w}C_{p,w}(T_{t,w}-T_{\mathrm{ini},w})$$

(3) $$CCF=\frac{Q_{\mathrm{use}}}{m_{w}c_{p.w}(T_{\max,w}-T_{\mathrm{ini},w})+m_{P}L_{P}+m_{P}c_{p,P}(T_{\max,P}-T_{\mathrm{ini},P})+m_{\mathrm{Ba}}L_{\mathrm{Ba}}+m_{\mathrm{Ba}}c_{p,\mathrm{Ba}}(T_{\max,\mathrm{Ba}}-T_{\mathrm{ini},\mathrm{Ba}})}$$

For the energy stored in the tank $Q_{\mathrm{ch}\arg e}$, it can be expressed as Equation (4), while Equation (5) shows the calculation of the energy storage efficiency ξ of the system. The energy storage efficiency demonstrates the actual operational effectiveness of the heat storage system and measures how well a PCM retains energy for later use.

(4) $$Q_{\mathrm{charge}}=m_{w}c_{p,w}(T_{t,w}-T_{\mathrm{ini},w})+m_{P}L_{P}+m_{P}c_{p,P}(T_{t,P}-T_{\mathrm{ini},P})+m_{\mathrm{Ba}}L_{\mathrm{Ba}}+m_{\mathrm{Ba}}c_{p,\mathrm{Ba}}(T_{t,\mathrm{Ba}}-T_{\mathrm{ini},\mathrm{Ba}})$$

(5) $$\xi =\frac{Q_{\mathrm{charge},\mathrm{PCM}}}{Q_{\mathrm{charge},w}}$$

where $Q_{\mathrm{charge},\mathrm{PCM}}$ denotes the energy stored in the energy storage process in the system with PCM. $Q_{\mathrm{charge},w}$ denotes the energy stored in the whole system when there is only water. $m_{w}$ is the mass of water in the system, $m_{P}$ is the mass of paraffin in the system, $m_{\mathrm{Ba}}$ is the mass of BHOH in the system. $C_{p,w}$ is the specific heat of water, $C_{p,P}$ is the specific heat of the paraffin, $C_{p,\mathrm{Ba}}$ is the specific heat of the BHOH. *L*_P_ represents the latent heat of paraffin. *L*_Ba_ represents the latent heat of BHOH.

*T*_max,w_ is the maximum value of water temperature in the system, *T*_max,P_ is the maximum value of paraffin temperature in the system; and *T*_max,Ba_ is the maximum value of BHOH temperature in the system. *T_t_*_,w_ is the temperature of the water at a given time, and *T_t,_* _P_ is the temperature of the paraffin at a given time, *T_t_*_,Ba_ is the temperature of the BHOH at a given time. *T*_ini,w_ is the initial temperature of the water. *T*_ini,P_ is the initial temperature of the paraffin. *T*_ini,w_ is the initial temperature of the BHOH. *T*_0_ indicates the ambient temperature, which is 22 °C.

### 3.2. Exergy Analysis

Energy analysis cannot assess the depreciation and loss of energy mass in a system, leaving the nature of energy loss unexplained. In contrast, exergy analysis evaluates system performance based on energy mass, making exergy analysis a more scientific and comprehensive approach.

The exergy stored in PCM and the total exergy stored in the system during charging can be obtained as defined in [51]. Where, $Ex_{\mathrm{PCM}}$ is the exergy value stored in the PCM, $Ex_{\mathrm{total}}$ is the total exergy value stored in the system.

(6) $$Ex_{\mathrm{PCM}}=m_{P}[L_{P}+C_{p,P}((T_{t,P}-T_{ini,P})-T_{0}\ln\frac{T_{t,P}}{T_{\mathrm{ini},P}})]+m_{\mathrm{Ba}}[L_{\mathrm{Ba}}+C_{p,\mathrm{Ba}}((T_{t,\mathrm{Ba}}-T_{\mathrm{ini},\mathrm{Ba}})-T_{0}\ln\frac{T_{t,\mathrm{Ba}}}{T_{\mathrm{ini},\mathrm{Ba}}})]$$

(7) $$Ex_{\mathrm{total}}=m_{w}C_{p,w}[(T_{\max,w}-T_{\mathrm{ini},w})-T_{0}\ln\frac{T_{\max,w}}{T_{\mathrm{ini},w}}]$$

The exergy storage efficiency of the PCM during energy release is expressed in terms of η, which is given by the following equation formulated in [51].

(8) $$\eta =\frac{Ex_{\mathrm{PCM}}}{Ex_{\mathrm{total}}}\;$$

Furthermore, as indicated in Ref. [52], based on the exergy balance, the exergy destruction ($Ex_{d,\mathrm{PCM}}$) of the PCM during energy release can be determined using the following equation.

(9) $$Ex_{d,\mathrm{PCM}}=m_{P}[L_{P}+C_{p,P}(T_{t,P}-T_{\mathrm{ini},P})]\left(1-\frac{T_{0}}{T_{t,P}}\right)+m_{\mathrm{Ba}}[L_{\mathrm{Ba}}+C_{p,\mathrm{Ba}}(T_{t,\mathrm{Ba}}-T_{\mathrm{ini},\mathrm{Ba}})]\left(1-\frac{T_{0}}{T_{t,\mathrm{Ba}}}\right)$$

## 4. Results

As briefly mentioned earlier, in order to understand the energy storage characteristics of PCMs and to compare them with the combined use of multiple PCMs, experimental investigation has been undertaken to comprehend the energy storage characteristics of a phase change energy storage tank using two PCMs. The results of these two energy storage PCMs are elaborated in the following subsections.

### 4.1. Study of Energy Storage Properties of Paraffin

**Figure 8:**
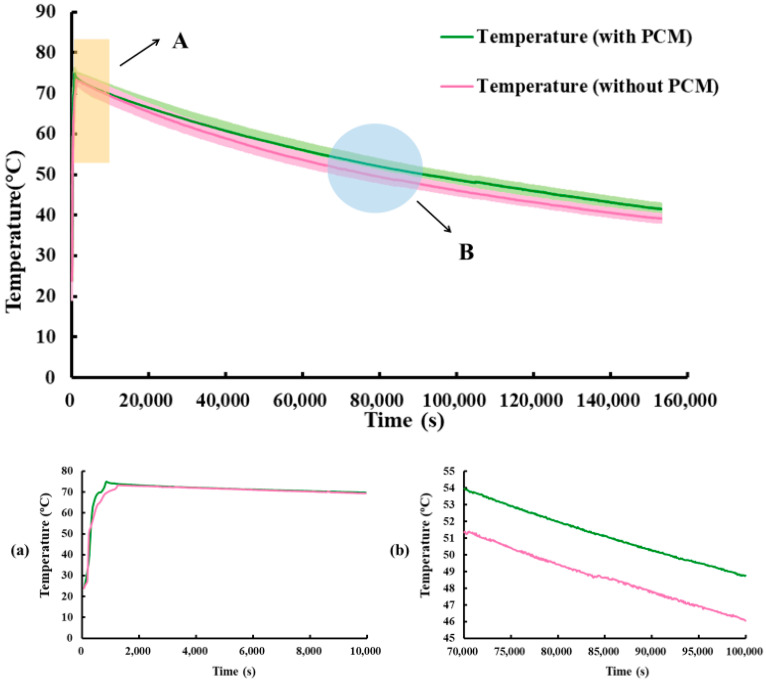
Water temperature variation curve of phase change energy storage tank with paraffin comprising 1.80% by mass only. (**a**) Enlarged view of position “A”, (**b**) Enlarged view of position “B”.

The exothermic process of the storage tank was analyzed by comparing the data with and without PCM (paraffin), as shown in Figure 8. In the early stage of the exothermic process, it was observed that the temperature changes in both cases were similar. However, as the temperature kept approaching the phase-change temperature of the paraffin (50 °C), the tank with the PCM and the tank without the PCM exhibited a maximum temperature difference. After that, the temperature changes were approximately the same, and furthermore, at the same time, the temperature of the tank with the PCM was higher than that of the tank without the PCM, by 2–3 °C.

### 4.2. Study of Energy Storage Properties of BHOH

**Figure 9:**
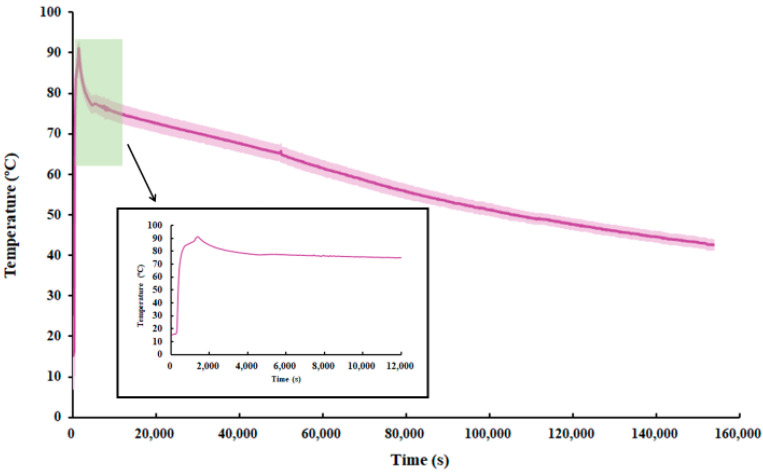
Temperature changes with time in the energy storage tank containing BHOH comprising 1.80% by mass only.

As depicted in Figure 9, during the exothermic process of temperature change in the energy storage tank containing BHOH, the temperature rises rapidly in the first 2000 s or so. This is evident from the steep slope of the temperature curve. The rapid temperature rise can be attributed to the influx of 90 °C hot water into the phase change storage tank when the temperature of the PCM is relatively low. At this stage, there exists a significant temperature difference between the incoming hot water and the PCM, resulting in intense heat transfer. Consequently, heat is primarily stored in the form of sensible heat during this phase. Upon reaching about 78 °C, which is the melting temperature of the PCM, the process transitions into a phase change stage. In related studies [49], it was discovered that the phase transition point of BHOH tends to change with an increase in phase transition times. Specifically, the temperature range observed was 72–78 °C, which is considered reasonable. This finding suggests that the phase transition behavior of BHOH can be influenced by factors such as duration or frequency of phase transitions. This phase change process is characterized by a quasi-steady state. Here, heat is predominantly stored in the form of latent heat until the PCM is completely melted. As the heat storage continues, the temperature of the PCM steadily increases. However, as the temperature difference between the PCM and the hot water in the tank decreases over time, the slope of the temperature curve gradually flattens. At around 50,000 s the temperature in the tank showed a small peak which is believed to be due to the minor fluctuation of the ambient temperature. The temperature also gradually decreases entering the holding process.

### 4.3. Characterization of Combined Energy Storage of Paraffin and BHOH

**Figure 10:**
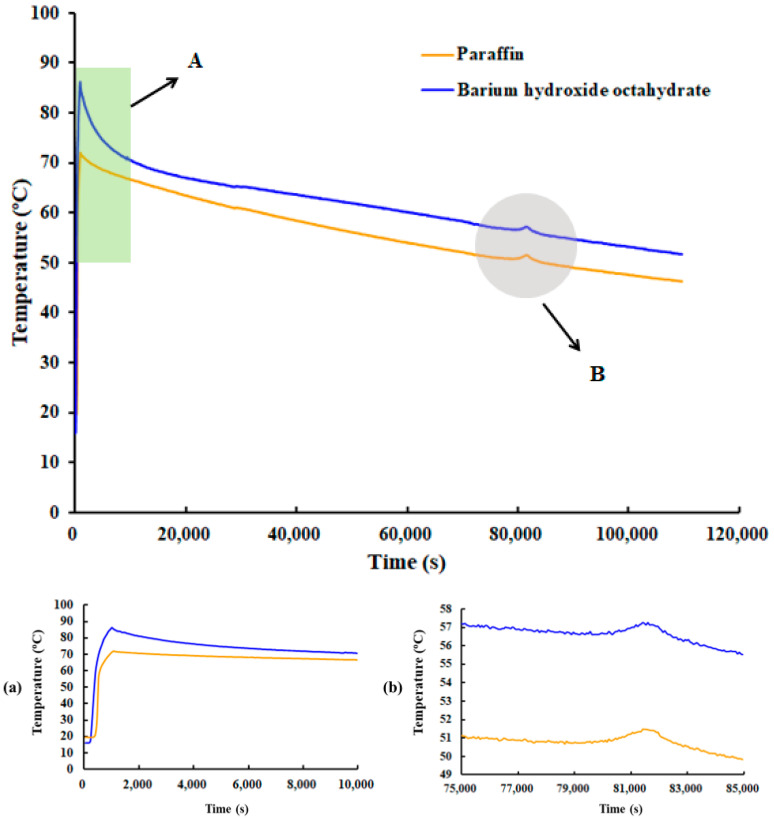
Temperature changes with time in the energy storage tank containing both paraffin comprising 1.75% by mass and BHOH comprising 5.12% by mass. (**a**) Enlarged view of position “A”, (**b**) Enlarged view of position “B”.

As illustrated in Figure 10, the exothermic process during the temperature change in the energy storage tank, which contains both paraffin and BHOH, shows a rapid increase in temperature within the first 2000 s. This is evident from the steep slope of the temperature curve in the figure. The rapid temperature rise can be attributed to the continuous infusion of hot water at 90 °C into the phase change energy storage tank. At this point, the temperature of the PCM is relatively low, creating a significant temperature difference between the incoming hot water and the PCM. This intensifies heat transfer, primarily through sensible heat storage. Once the temperature reaches approximately 78 °C, corresponding to the phase change temperature of BHOH, there is a noticeable plateau in the temperature change. Subsequently, the system enters the exothermic stage, characterized by a gradual decrease in temperature. Around the 80,000 s mark, the water temperature reaches approximately 50 °C. The system’s water temperature was observed to fluctuate, initially increasing and then decreasing, which indicates that the paraffin wax underwent a phase change. Following this, the entire system continued to exhibit exothermic behavior. Since the BHOH is located beneath the paraffin, it comes into contact with the hot water first, resulting in a slightly higher temperature than the paraffin.

### 4.4. Result of the Energy and Exergy Analysis

**Figure 11:**
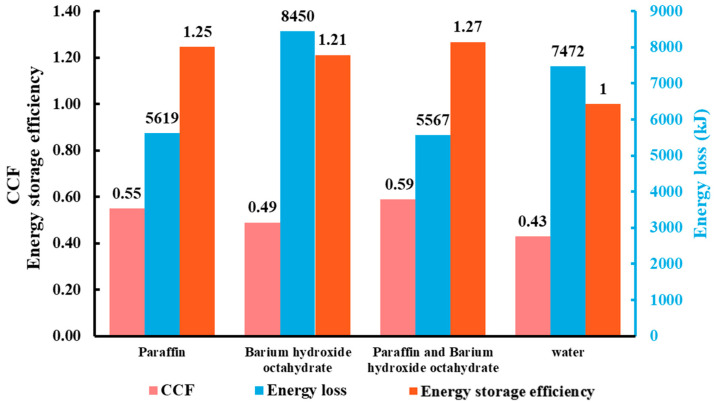
Cumulative Charge Fraction (CCF), energy loss, and energy storage efficiency for tanks with different PCMs.

Based on the above equations, the CCF, energy storage efficiency, and energy loss of the PCM have been determined and plotted in Figure 11. The figure illustrates the CCF, energy loss, and energy storage efficiency of different types of PCMs and without PCMs. The data reveals that systems using a combination of two PCMs achieve the highest CCF (0.59), lowest energy loss (5567 kJ), and highest energy storage efficiency improvement value (27%).

In addition, paraffin can significantly improve system performance in terms of energy storage, as evidenced by higher CCF and energy storage efficiency compared to systems without PCM. The CCF of BHOH is very close to that of the non-PCM system, and the energy loss of BHOH is highest. Probably due to its higher phase transition temperature, which can lead to premature release of latent heat. The energy storage efficiencies of the thermal storage systems using PCM are increased compared to water, at around 20–30%, due to the part of the energy stored in the PCM. Systems employing both PCMs also exhibit superior energy capacity relative to other configurations.

**Figure 12:**
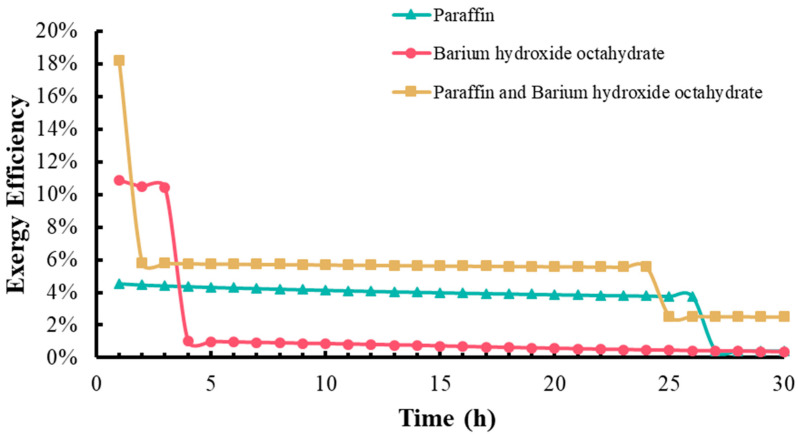
Exergy efficiency changes with time in the energy storage tank containing PCM.

As shown in Figure 12, the exergy efficiency hourly has been estimated over 30 h using the previously discussed equation. This data enabled us to plot a curve showing how exergy efficiency varies over time. The addition of PCMs to the water tank resulted in an increase in exergy efficiency across all experiments. Notably, when both PCMs were used together, the exergy efficiency peaked at 18%. Following the phase change of BHOH, however, the exergy efficiency dropped to 6% but remained stable for an extended period. In scenarios where only one PCM was used, BHOH led to a higher, albeit brief, spike in efficiency, whereas paraffin’s impact lasted longer. Combining both PCMs in the tank induced complex temperature dynamics, enhancing the exergy efficiency compared to using a single PCM. This suggests that the PCMs may have a synergistic effect, enhancing thermal storage across various temperatures. The exergy destruction calculated using Equation (9) yields the following values after 30 h: 147 kJ for paraffin alone, 480 kJ for BHOH alone, and 641 kJ for their combined use. Additionally, the calculated exergy efficiency represents the ratio of the exergy stored in the PCM during the exothermic process to the total exergy stored in the system, rather than the overall exergy efficiency of the complete cyclic process.

## 5. Discussion

This study investigated an energy storage tank with two PCMs (paraffin and BHOH). The experimental study revealed that a single PCM (BHOH) and a dual PCM system (paraffin and BHOH) kept the temperature stable for longer. Comparing situations with and without PCM (paraffin), it is clear that when PCM is added, the temperature drops much more slowly, by a difference of 2 to 3 °C after the phase change point. Also, when both PCMs were added to the storage tanks at the same time, there was a clear temperature recovery for about 2000 s around the paraffin phase transition temperature, demonstrating the release of stored thermal energy from the PCMs.

The combination of the two PCMs has the highest cumulative change fraction (CCF) and energy storage efficiency, as well as low energy loss. Using paraffin as a PCM not only enhances the system’s energy storage performance but also increases its energy capacity and storage efficiency. When both PCMs are used, the system’s energy capacity surpasses that of the other cases.

The integration of PCMs into the energy storage system notably enhances the exergy efficiency, achieving peaks as high as 18%. When considering the individual effects of PCMs, BHOH demonstrates a swift but transient increase in exergy efficiency. In contrast, paraffin shows a more sustained efficiency over an extended period. Introducing both PCMs into the tank results in intricate temperature dynamics due to their interaction, which not only improves the exergy efficiency compared to using a single PCM but also exhibits effective thermal storage across various temperatures. This points to a synergistic effect arising from the combined phase change heat of the PCMs, thereby optimizing the energy storage system’s overall performance. The selection and optimization of PCMs can be tailored to specific weather conditions and energy needs, providing wider energy storage solutions. Additionally, based on Ref. [53], the heat transfer coefficients between PCM and water are approximately 6.2 W/(m^2^·K) for paraffin and 37.2 W/(m^2^·K) for BHOH, respectively.

The findings offer valuable insights for intermittent renewable energy storage and application. Improving solar energy utilization efficiency and refining energy storage technologies can accelerate clean energy adoption, aiding in the pursuit of sustainable development goals. The findings of this study can expand the current understanding of PCMs for energy storage and provide important insights for designing more efficient energy storage systems.

Several factors, including climatic conditions, thermophysical properties of PCMs, design of the LHS tanks, and variability in solar energy capture and heat loss from both the piping and storage tanks, influence the efficiency of thermal storage systems, as demonstrated by this study and prior studies. Additionally, water circulation within the system and differing PCM levels have an impact on performance. In light of these factors, this study proposes areas for future research, such as enhancing LHS tank designs and assessing thermal storage systems from both technical and economic perspectives under various climatic and seasonal conditions.

### Implication

The study further enhances the awareness of technical readers, policymakers, investors, and researchers by introducing a novel perspective on energy optimization and environmental protection. Extended research is needed to better understand how different combinations of PCMs store thermal energy and how they can be optimized for real-world applications to meet diverse energy storage requirements.

## 6. Conclusions

This study combined two phase change materials, paraffin and BHOH, with a phase change energy storage tank to enhance thermal energy storage performance. This study included an energy and exergy analysis of the two PCMs used in medium-temperature thermal energy storage systems. The main conclusions of this study are summarized as follows:

- Single-phase change material (BHOH) and dual-phase change material systems (paraffin and BHOH) demonstrated extended temperature stability. After the phase transition, the addition of paraffin reduced the temperature drop by 2–3 °C.
- The dual phase change material (PCM) system exhibited notable temperature recovery for approximately 2000 s around the paraffin phase transition temperature, indicating effective thermal energy release. The combination of PCMs achieved the highest cumulative charge fraction (CCF) and energy storage efficiency, as well as the lowest energy loss.
- Using both PCMs together increased exergy efficiency, reaching up to 18%. While BHOH showed a transient boost in exergy efficiency, paraffin maintained a more sustained performance.
- The synergistic effect of the dual PCMs optimizes thermal storage and efficiency across a variety of temperatures, providing tailored energy storage solutions.
- Future research should explore the use of various PCMs to identify combinations that are both efficient and cost-effective. Additionally, the design of LHS tanks and the performance of thermal storage systems can be assessed from. both technical and economic perspectives across different climatic and seasonal contexts.

## Figures and Tables

**Table 1:** Thermal properties of some common inorganic salt hydrate PCMs (NA means not available) [25,26,27,28].

Material	Melting Point (°C)	Latent Heat (J/g)	Specific Heat (Solid/Liquid) (kJ/(kg·°C))	Thermal Conductivity (Solid/Liquid) (W/(m·°C))	Density (Solid/Liquid) (g/cm^3^)
LiNO_3_·3H_2_O	30	296	1.73/2.76	0.82/0.584	1.575/1.425
LiClO_3_·3H_2_O	8	155	NA/NA	NA/NA	1.72/1.53
Na_2_SO_4_·10H_2_O	32	254	NA/NA	NA/NA	NA/NA
Na_2_S_2_O_3_·5H_2_O	48–55	187, 209	1.46/2.38	NA/NA	1.75/1.67
Na_2_HPO_4_·12H_2_O	35–44	280	1.70/1.95	0.514/0.476	1.522/1.442
CaCl_2_·6H_2_O	29.3	171	1.42/2.10	1.088/0.54	1.71/1.562
CH_3_COONa·3H_2_O	58	226	1.87/3.35	NA/NA	1.45/1.28
Ba(OH)_2_·8H_2_O	78	267, 301	2.18/NA	1.17/NA	1.2/NA
Mg(NO_3_)_2_·6H_2_O	89, 90	149, 163	1.81/2.48	0.669/0.49	1.636/1.550
MgCl_2_·6H_2_O	117	165, 169	2.25/2.61	0.707/0.570	1.569/1.45

**Table 2:** Literature on use of PCM in solar energy systems.

Author	Findings	Knowledge Gaps
Palacio et al. [33]	The addition of PCM (paraffin) improves the performance of solar collectors. Two consecutive days of measurements were considered for case to study the PCM behavior during nocturnal periods and to observe if the results of first day may affect the next one. The presence of PCM has a significant effect on the performance of solar collectors during daytime and nighttime hours.	No in-depth discussion of the type, amount, and shape of PCMs and other performance metrics of solar collectors.
Varol et al. [34]	The experiments were performed in Elazig, Turkey, where latitude, 38.41° N, longitude, 39.14° E, and altitude 1067 m above sea level. Solar collectors using sodium carbonate decahydrate (Na_2_CO_3_·10H_2_O) are more efficient than conventional systems in collecting hot water in winter. Na_2_CO_3_·10H_2_O has a melting point of 306 K and a latent heat of 267 kJ/kg. Vector machine technique. performs best in predicting the performance of solar collectors.	No consideration was given to collector main component temperatures, heat transfer rates, PCM main component temperatures, heat transfer rates, internal behaviors, night-time operation, or the number of consecutive days.
Pawar and Sobhansarbandi [35]	Heat pipe evacuated tube solar collectors (HPETCs) system with integrated tritriacontane (C_33_H_68_) + Cu porous metal improves peak temperature by nearly 21 °C and achieves energy efficiency of up to 85.64%, reducing hot water production costs.	There is no discussion of the challenges and limitations that HPETC systems integrating PCM + Cu porous metals may face in practical applications.
Zhou et al. [36]	PCM-antifreeze solar thermal systems have better performance in cold weather conditions and can effectively prevent system freezing. Operating in conditions of total solar energy of 13.74 MJ/m^2^, ambient temperature −5 °C.	Latent heat devices for solar collectors are still in the research phase, evaluating the performance of PCMs as frost protection systems rather than energy characteristics.
Assareh et al. [37]	Increasing the inner diameter of the tubes in the collector system increases the energy discharge time of the PCM. As a result, the thermal accessibility at night will be extended. Increasing the tube diameter while keeping the system conditions constant allows for a higher energy storage capacity in the PCM.	There is no clear indication of the limitations and challenges of the method in practical application, nor is a comparison with other optimization methods provided.
Nekoonam and Ghasempour [38]	50% increase (decrease) of the HTF (heat transfer fluid) flow rate results in 12% increase (25% decrease) in the charging required time. PCM density and thermal melting temperature have a significant effect on energy storage capacity. Thermal capacity has a significant effect on the level of storage in the energy storage unit during charging.	Lack of comparative analyses of different types of solar storage systems and examples of practical applications.
Yeh et al. [39]	Effectiveness of salt hydrate based composite PCM, SAT (CH_3_COONa·3H_2_O)-SPM (NaH_2_PO_4_·H_2_O)-EG (expanded graphite), for thermal charging and discharging. Effective thermal performance of PCM improves energy storage efficiency of solar thermal collectors.	The experiment only tested a single shape of PCM and did not consider the use of multiple shapes of PCM in combination.
Luo et al. [40]	Experiments were conducted in Suqian City, Jiangsu Province, China, to test a novel air-based dual-channel solar collector embedded with phase change materials (based on aluminum ammonium sulfate dodecahydrate mixed with other inorganic salt phase change materials). The phase change material was able to mitigate the change in outlet temperature during fluctuations in radiation intensity, with the outlet temperature decreasing by only 7.5 K even when the radiation intensity decreased by 39% in 30 min. In addition, the PCM was able to continuously release heat during the night, extending the operating time of the collector by 8 h. The PCM was also able to reduce the heat loss during the night, which resulted in a decrease in the outlet temperature of the solar collector. Solar collectors embedded with phase change materials show good potential in terms of thermal efficiency and stability.	Experimental tests are conducted under specific environmental conditions and may not be fully representative of actual performance under different climatic and environmental conditions. This limits the general applicability of the results.
Narayanan et al. [41]	Paraffin, oleic acid, and 0.5wt% nano-graphite were used as phase change materials for testing on solar collector systems. The findings of the system demonstrate that the use of PCM nanocomposites increases the charging rate of PCM while decreasing the rate of heat discharge by PCM to water, thus increasing the maximum use of solar energy and thus the efficiency of the system.	The effect of different mass fractions of nano-graphite on the performance of novel eutectic phase change material and on solar water heaters was not explored.

**Table 3:** Properties of Two Selected Phase Change Materials [25,49].

Material	Paraffin	Ba(OH)_2_·8H_2_O (Barium Hydroxide Octahydrate)
Melting point (°C)	50–52	78
Density (g/cm^3^)	0.9	2.18
Specific heat (kJ/(kg·°C))	2.95	1.17
Latent heat (kJ/(kg·°C))	189	267
Thermal conductivity (W/(m·°C))	0.2	1.2

## Data Availability

The raw data supporting the conclusions of this article will be made available by the authors on request.
